# Chemical Composition and Antioxidant Potential of Five Algae Cultivated in Fully Controlled Closed Systems

**DOI:** 10.3390/molecules28124588

**Published:** 2023-06-06

**Authors:** Paulo Nova, Ana Pimenta-Martins, Élia Maricato, Cláudia Nunes, Helena Abreu, Manuel A. Coimbra, Ana Cristina Freitas, Ana Maria Gomes

**Affiliations:** 1 CBQF—Centro de Biotecnologia e Química Fina—Laboratório Associado, Escola Superior de Biotecnologia, Universidade Católica Portuguesa, Rua Diogo Botelho 1327, 4169-005 Porto, Portugal; pnova@ucp.pt (P.N.); apimenta@ucp.pt (A.P.-M.); afreitas@porto.ucp.pt (A.C.F.); 2 LAQV/REQUIMTE, Department of Chemistry, University of Aveiro, 3810-193 Aveiro, Portugal; elia@ua.pt (É.M.); mac@ua.pt (M.A.C.); 3 CICECO—Aveiro Institute of Materials, Department of Materials and Ceramic Engineering, University of Aveiro, 3810-193 Aveiro, Portugal; claudianunes@ua.pt; 4 AlgaPlus, Travessa Alexandre da Conceição s/n, 3830-196 Ílhavo, Portugal; helena.abreu@algaplus.pt

**Keywords:** seaweeds, algae, chemical composition, antioxidant characterization, fully controlled closed systems

## Abstract

In this study, the chemical composition and antioxidant profile of five edible macroalgae, *Fucus vesiculosus*, *Palmaria palmata*, *Porphyra dioica*, *Ulva rigida*, and *Gracilaria gracilis*, cultivated in fully controlled closed systems, were determined. Protein, carbohydrates, and fat contents ranged between 12.4% and 41.8%, 27.6% and 42.0%, and 0.1% and 3.4%, respectively. The tested seaweeds presented considerable amounts of Ca, Mg, K, Mn, and Fe, which reinforce their favorable nutritional profile. Regarding their polysaccharide composition, *Gracilaria gracilis* and *Porphyra dioica* were rich in sugars common to agar-producing red algae, and *Fucus vesiculosus* was composed mainly of uronic acids, mannose, and fucose, characteristic of alginate and fucoidans, whereas rhamnose and uronic acid, characteristic of ulvans, predominated in *Ulva rigida*. Comparatively, the brown *F. vesiculosus* clearly stood out, presenting a high polysaccharide content rich in fucoidans, and higher total phenolic content and antioxidant scavenging activity, determined by DPPH and ABTS. The remarkable potential of these marine macroalgae makes them excellent ingredients for a wide range of health, food, and industrial applications.

## 1. Introduction

Algae are photosynthetic eukaryotes that play a crucial role in the ecosystem, possessing a diverse biology and rich evolutionary history with important participation in the shaping of the planet’s atmosphere, and even nowadays the production of oxygen by algae is about 50% of all oxygen produced [1,2,3]. Seaweeds have been on earth far beyond the dawn of mankind and have had varying degrees of influence on human societies across history [2]. Nowadays, scientific data are opening the path for the full valorization of algae, showing their remarkable potential for a wide range of health and industrial applications [4,5].

The marine environment is an untapped source of unique and efficient compounds, where algae stand out as a valuable source of chemical compounds such as polysaccharides, proteins, minerals, enzymes, glycoproteins, polyunsaturated fatty acids, sulfolipids, phenolics, terpenoids, and other secondary metabolites [6,7,8]. Seaweed compounds have the advantages of being of natural origin and, given their antioxidant, antibacterial, anticoagulant, and antitumor activities, their ingestion may impact positively the health of individuals and reduce the risk of developing certain pathologies such as cancer, type 2 diabetes, cardiovascular diseases and related risk factors, and neurological diseases, among others [8,9,10,11,12,13,14].

Because of their remarkable advantages, macroalgae are excellent for use as ingredients in the development of innovative functional foods, dietary supplements, nutraceuticals, and even pharmaceuticals. In fact, in the year 2017, the algae product market was valued at USD 3.78 billion, being expected to achieve a value of USD 5.17 billion by 2023 [15,16,17]. This clearly highlights increasing consumer demand for foods and algae-derived products that combine natural and sustainable ingredients, high nutritional value, and health benefits [4,16,18,19].

Algae grow by adapting to environmental stress conditions such as sunlight, salinity, temperature, carbon dioxide supply (CO_2_), and others [20]. Because environmental and seasonal conditions are extremely variable and typically very harsh, wild algae’s chemical composition diverges greatly even within the same species [20,21]. Cultivation of algae in fully controlled systems offers the advantage of controlling development and processing conditions, creating a sustainable biomass product with controlled quality and biological properties [22]. This type of production provides algae biomass with improved quality standards, higher productivity, and ensures that the culture is not contaminated by undesirable microorganisms [22]. Furthermore, farmed seaweed contributes to sustainability development goals, since it does not require great agricultural areas, as plant-based proteins do, and may be used directly as an ingredient for food, feed, or supplement applications.

Characterization of seaweed in terms of chemical compounds and biological activities is an essential step required for the validation and valorization of algae for further nutritional, biotechnological, medical, and industrial applications. Therefore, the main objective of the present study was to determine the chemical composition and antioxidant activity of five different edible species of algae produced in fully controlled closed systems located on the western coast of the Iberian Peninsula: *Fucus vesiculosus* (brown algae), *Palmaria palmata*, *Porphyra dioica*, *Gracilaria gracilis* (red algae), *and Ulva rigida* (green algae). This study strongly contributes to generating an open mapping of land-based, integrated multi-trophic aquaculture (IMTA)-farmed seaweed composition, all edible and with tremendous potential for health, food, industrial, and biotechnological applications.

## 2. Results and Discussion

### 2.1. Proximate Composition 

The chemical profile of the five previously dried farmed seaweeds under analysis is presented in Table 1. The contrasting profiles that are observed for the different seaweeds highlight the different taxonomic and physiological characteristics that are being covered with specimens from all three main algae groups. 

Algae biomass presents high water content, which constitutes a good environment for enzymatic activity, and microbial growth, which leads to high perishability [23]. Drying of these marine ingredients is an excellent strategy to further develop industrial, pharmaceutical, and biotechnological applications. This process consists of removing water and consequently reducing moisture, leading to product stabilization and longer shelf life [23]. Despite having undergone a similar drying process, the seaweed specimens varied significantly (*p* < 0.05) in their final moisture contents, which ranged from 3.2 g/100 g _dry seaweed_ in the red seaweed *P. palmata*, followed closely by *G. gracilis* (4.1 g/100 g _dry seaweed_), to 16.2 g/100 g _dry seaweed_ in the green seaweed *U. rigida*. The brown macroalgae *F. vesiculosus* presented an intermediate moisture content of 12.4 g/100 g _dry seaweed_ (Table 1).

Algae are considered a valuable source of protein, yet their contents vary according to species and farming conditions. Protein content was statistically different among all species (*p* < 0.05). The brown algae *F. vesiculosus* revealed contents of 12.4 g/100 g _dry seaweed_, followed by 19.5 g/100 g _dry seaweed_ in the green alga *U. rigida*, and the three highest values (two- to three-fold higher) were registered in the red algae species (highest value of 41.8 g/100 g _dry seaweed_ in *P. Palmata)*. These results are consistent with the results found in scientific literature. Studies performed by Wells et al. [19] reported that red and green algae generally presented higher protein levels compared to brown algae, and Cherry et al. (2019) in their review reported protein content ranges consistent with those found in this study, namely, from 0.67% to 45.0% in red seaweeds, from 5.02% to 19.66% in brown seaweeds, and from 3.42% to 29.80% in green seaweeds [18,19]. Such variability is understandable since harvested seaweeds derive from different growth process (cultivation versus wild collection), lifecycle, seasonality (temperature and light intensity), geographical distribution, and ecological conditions (available nutrients, salinity). All such parameters have a great impact on the chemical composition of seaweed, including protein content. Notably, the red seaweeds in the present study, which derived from integrated multi-trophic aquaculture systems, where growth is performed under controlled conditions, presented higher protein values than those previously described by Rodrigues et al. (2015) for wild *G. gracilis* (20.2%) harvested in Buarcos bay on the Central West Coast of Portugal, by Fernández-Segovia, Lerma-García, Fuentes, and Barat (2018) for *P. dioica* (approximately 22%), collected in the Atlantic coastal region of Galicia (Spain), and by Bjarnadóttir et al. (2018) for *P. Palmata* (24.8%) harvested in Skjerstadfjorden, Bodø, Norway [13,24,25]. In contrast, protein values are below those previously described by Gadberry et al. (2018) for *U. rigida* (29.7%) produced in land-based cultivation systems, and yet almost equivalent to those presented by Lorenzo et al. (2017) for *F. vesiculosus* (12.99%) collected in the Galician coast (Spain) [13,24,25,26,27]. 

The macroalgae studied in the present work, especially *G. gracilis*, *P. dioica*, and *P. palmata* (red species), are excellent sources of alternative high-quality protein with levels comparable to those present in meat, eggs, soybean, or milk [28]. Therefore, these organisms could be part of the solution for the need for alternative protein sources and imperative increase in food production by around 70%, in order to meet the demands of a growing population that is expected to increase 2.3 billion people by 2050 [28]. Seaweed production has nutritional and productivity effectiveness advantages in comparison with traditional high-protein crops such as soybean and pulse legumes [28,29]. Macroalgae can develop without the need for fresh water or arable land and possess a higher protein yield per unit area in comparison with terrestrial crops (2.5–7.5 tons/Ha/year in comparison with 0.6–1.2 tons/Ha/year and 1–2 tons/Ha/year, for soybean and pulse legumes, respectively) [28,29]. In short, seaweeds are definitely a good source of high-quality and sustainably produced protein that should be implemented in healthy diets and in the development of novel food products in the near future [16]. 

Total carbohydrate content ranged from 9.3 g/100 g _dry seaweed_ in the red seaweed *P. palmata* to 30.2 g/100 g _dry seaweed_ in the brown algae *F. vesiculosus.* The proximate composition of this macronutrient varied greatly among species, with higher values being found in the brown and green species analyzed (30.2 g/100 g and 27.9 g/100 g _dry seaweed_, respectively). Among the red algae, *P. dioica* and *G. gracilis* presented very similar values (29.8 and 26.8 g/100 g _dry seaweed_), and *P. palmata* the lowest value among seaweed species (9.3 g/100 g _dry seaweed_) (Table 1). Carbohydrate composition of seaweeds includes important and biologically active molecules, including polysaccharides [30], a source of dietary fibers, whose characterization will be presented and discussed in the following sections. Dietary fibers are important nutritional compounds since they cannot be completely degraded by human digestive enzymes, with benefits for human gut microbiota and overall health. 

Fat content in seaweeds is naturally low, and those analyzed in this study were no exception. The fat content of our macroalgae ranged from 0.078 g/100 g _dry seaweed_ in the green alga *U. rigida* to 3.4 g/100 g _dry seaweed_ in the brown alga *F. vesiculosus.* Among the red seaweed species assessed, *P. dioica* registered the highest total fat values (1.59 g/100 g _dry seaweed_). Results were statistically significantly different among all seaweeds except for comparisons between *G. gracilis* and *P. palmata* (red seaweed) and *P. palmata* and *U. rigida* (red and green seaweeds) (Table 1). Given their high nutritional value, and low-calorie and low-fat profiles, algae are an excellent asset to be incorporated into healthy balanced diets [31]. Furthermore, their versatility of use enables them to be consumed dried, fresh, pickled, cooked, or as a component of food products such as healthy snacks, soups, and bread, among others, which corroborates even further the potential of these organisms to be included in the daily diet of consumers [16,31]. The results obtained in the present research are quite similar to those obtained by Rodrigues et al. (2015) for *G. gracilis* collected from Buarcos bay on the Central West Coast of Portugal (0.60 g/100 g _dry seaweed_) and Lorenzo et al. (2017) for *F. vesiculosus* (3.75 g/100 g _dry seaweed_) collected on the Galician coast (Spain) [13,27]. In contrast, Gadberry et al. (2018) obtained higher lipid contents for *U. rigida* (2.97 g/100 g _dry seaweed_) produced in land-based cultivation systems, as well as Mæhre, Malde, Eilertsen, and Elvevoll (2014) for *P. palmata* (1.4 g/100 g _dry seaweed_) harvested off the coast of Norway [26,32]. Lower lipid contents were described by Fernández-Segovia et al. (2018) for *P. dioica* (0.08 g/100 g _dry seaweed_), harvested in Skjerstadfjorden, Bodø, Norway [24]. 

Ash contents were quite similar among the tested seaweeds, ranging from 23.41 g/100 g _dry seaweed_ for the red macroalgae *P. dioica* to 29.8 g/100 g _dry seaweed_ in the brown algae *F. vesiculosus.* Statistically significant differences were registered between all species for this parameter, except for comparisons between *F. vesiculosus* and *G. gracilis* and between *P. palmata* and *U. rigida* (Table 1). Our results were quite similar to the results obtained by Rodrigues et al. (2015) for *G. gracilis* (24.8 g/100 g _dry seaweed_) and by Fernández-Segovia et al. (2018) for *P. dioica* (24 g/100 g _dry seaweed_) [13,24]. In contrast, Lorenzo et al. (2017) obtained lower results in comparison with our analysis for *F. vesiculosus* (20.7/100 g _dry seaweed_) and Gadberry et al. (2018) and Mæhre et al. (2014) for *U. rigida* (32 g/100 g _dry seaweed_) and *P. palmata*, respectively [26,27,32]. These differences in ash contents among the same species were probably due to different growing conditions such as sunlight, salinity, temperature, and carbon dioxide supply (CO_2_), among others [20]. In fact, all algae samples from these previous studies were harvested from the wild, with the exception of those performed by Gadberry et al. (2018), in which algae were also grown in land-based cultivation systems. In this case, it should be highlighted that growth conditions greatly varied from those used for the algae studied herein [26]. 

### 2.2. Elemental Composition of Seaweeds 

Minerals are essential for the human body. As such, their adequate dietary intake greatly contributes to the prevention of chronic and degenerative diseases such as cancer, neurological, and cardiovascular diseases and premature aging [33,34,35]. In addition to their macronutrient value, algae can also deliver a considerable amount of highly valuable elements, which reinforces their potential to be incorporated into the development of functional foods, pharmaceuticals, and nutraceuticals [5,36]. Seaweed mineral content is very variable and is tremendously influenced by several exogenous and endogenous factors such as environmental conditions, morphological features, and geographic location [37,38]. Among the tested five seaweed species, the most representative micronutrients detected were calcium (Ca), potassium (K), sodium (Na), magnesium (Mg), iron (Fe), and manganese (Mn). Phosphorus (P) and zinc (Zn) were consistently present in all the seaweed species, but in lower amounts in comparison with the previously mentioned elements. The full results are shown in Table 2. 

Sodium was the most representative mineral in overall quantitative terms for *F. vesiculosus* and *P. dioica* (47.64 and 42.56 mg/g _dry seaweed_, respectively), and values differed statistically (*p* < 0.05) between all algae species. Furthermore, brown and red algae species presented higher amounts of Na compared to the green algae (Table 2). Regarding K content, this was the most abundant element present in the *G. gracilis*, *P. palmata* (red species), and *U. rigida* (green species) seaweeds, ranging from 13.64 mg/g _dry seaweed_ in *U. rigida* to 89.20 mg/g _dry seaweed_ in the red seaweed *G. gracilis.* Additionally, K contents were statistically different among all species (*p* < 0.05), except for comparisons between *F. vesiculosus* (brown algae) and one of the red seaweed species, *P. palmata* (Table 2). High Na intake is closely related with high blood pressure (hypertension), a primary risk factor for cardiovascular disease, and as such a diet low in Na and high in K is widely recommended as an effective preventive strategy [39,40]. Several studies have established that Na-to-K intake ratio (Na:K) is a more relevant predictor of hypertension than the analysis of each of these micronutrients alone [39,40,41]. Foods with molar ratios of Na:K close to 1.0 are considered beneficial for human health, and as such their consumption should be privileged [42]. Regarding the tested seaweeds, *G. gracilis* and *P. palmata* (red species) presented Na:K ratios below 1.0, (0.50 and 0.85, respectively), whereas *F. vesiculosus*, *P. dioica*, and *U. rigida* obtained ratios above 1.0 (2.19, 2.52, and 1.31, respectively). Although *F. vesiculosus*, *P. dioica*, and *U. rigida* presented higher Na:K ratios, these results are still below the results registered for cheese (4.94), preserved and tinned fish (3.64), processed meat (2.77), and pizza, crackers, and other salty snacks (3.31) [43]. Furthermore, the salty taste of *G. gracilis* and *P. palmata* makes these marine ingredients even more suitable candidates for salt substitutes to be used in the reformulation of different food categories where salt reduction means not only is Na intake reduced, but K intake is enhanced together with that of other important minerals [13,16]. 

Calcium values were statistically different among all species (*p* < 0.05), and it was within the red algae species group that the minimum and maximum values were reported, ranging from 2.25 mg/g _dry seaweed_ in *P. dioica* to 15.91 mg/g _dry seaweed_ in *P. palmata*. Between the brown and green algae species, *F. vesiculosus* (brown algae) registered more than double the Ca content of *U. rigida* (green algae) (Table 2). This mineral is the most prevalent in the human body and its adequate intake is closely related to bone mineral density [44,45]. Furthermore, recent randomized controlled trials have also established a relationship between Ca intake and a reduced risk of high blood pressure, preeclampsia, or colorectal adenomas [44]. Macroalgae in the present study deliver good amounts of Ca that should be further explored in the development of alternative Ca supplements in the future targeting osteoporosis prevention and other low-Ca-intake-related diseases. Furthermore, macroalgae Ca content is higher than that of oranges, carrots, potatoes, and apples [38]. Regarding this mineral’s bioavailability in algae, it widely varies depending on the macroalgae species, food matrix, and other factors [5,46]. For example, the macroalgae *L. digitata* is rich in alginates that retain divalent cations, which include Ca, leading to poor bioavailability [38]. The same is expected to occur with *F. vesiculosus*, also a rich source of alginate [38]. On the other hand, the bioavailability of AAA Ca, a calcium supplement in oyster shell and the seaweed *Sargassum fusiformis*, has been reported to be very high [38].

Magnesium is an essential element for the human body, being a cofactor for more than 300 metabolic reactions such as DNA and RNA synthesis, protein synthesis, and cellular energy production and storage, among many others [47]. Furthermore, Mg is extremely important for overall health and its deficiency is closely related with the development of several chronic diseases such as neurological conditions, cerebrovascular accidents, and migraine headaches [47,48,49]. Magnesium concentrations ranged from 2.22 mg/g _dry seaweed_ in the red macroalgae *P. palmata* to 18.60 mg/g _dry seaweed_ in the green algae *U. rigida*, more than double the amount found in the red and brown species groups (Table 2). Notably, Mg concentrations were statistically similar (*p* > 0.05) among species from the same group (*Rhodophyta*) but statistically different (*p* < 0.05) between species from different groups, except for those reported for *P. dioica* and *F. vesiculosus* (red and brown algae, respectively). High Ca-to-Mg-intake ratios have been associated with increased risk of cardiovascular disease, colorectal and prostate cancer, and overall cancer mortality [50,51]. Current evidence suggests that a 1.70 to 2.60 range of Ca:Mg ratio can lead to reduction in disease risk [51]. However, this evidence is still limited because only a few studies have analyzed dietary Ca:Mg ratios <1.70 as well as >2.60 [51]. Furthermore, these benefits may be dependent on specific health outcomes and gender [51]. Macroalgae from the present study presented Ca:Mg ratios that ranged from 0.19 in the green algae *U. rigida* to 7.16 in the red algae *P. palmata* (Table 2). None of the five studied macroalgae are within the optimum Ca:Mg range of 1.70–2.60, and as such they should be incorporated into food matrixes that can help to balance Ca:Mg ratio. 

The macroalgae species tested herein were shown to be an important source of several trace elements. Regarding recommended dietary intake (RDI) contribution, Fe and Mn amounts were the most relevant across all the species tested. Values for Fe ranged from 0.46 mg/g _dry seaweed_ in *P. palmata* to 1.65 mg/g _dry seaweed_ in *G. gracilis.* This means that a consumption of 10 g of *G. gracilis provides* 117.9% of the RDI of this micronutrient, and as such, this seaweed is an excellent choice for consumers. This considerable amount of Fe can be extremely useful to incorporate into plant-based diets targeting specific dietary habits such as vegetarians and vegans, who generally have a low intake of this micronutrient [52]. Manganese values ranged from 0.06 mg/g _dry seaweed_ in the green seaweed *U. rigida* to 0.29 mg/g _dry seaweed_ in the brown seaweed *F. vesiculosus*. Interestingly, four out of the five algae tested presented Mn amounts very near or even above the RDI for this micronutrient. In particular, a consumption of 10 g of *F. vesiculosus* or *G. gracilis* provides 145.0% and 120.0% RDI, respectively (Table 2). Manganese insufficient dietary intake leads to several health problems such as poor bone formation and skeletal defects, altered carbohydrate and lipid metabolism, or abnormal glucose tolerance. Nevertheless, the deficiency in this micronutrient is extremely rare due to several available dietary sources, and as such it has only been reported under experimental settings [53]. Although the seaweed species analyzed herein have considerable amounts of Mn, their consumption is unlikely to impose any danger for consumers. The tolerable upper intake level for this micronutrient is 9–11 mg/day for adults and its absorption is tightly regulated in the gut—in fact, toxicity from dietary exposure has not yet been reported in the scientific literature [53]. 

### 2.3. Dietary Fiber Determination and Polysaccharide Characterization 

Diet composition, daily dietary intake, and acute dietary changes have a big impact on modulating the microbial composition of the gut [54]. In fact, scientists are currently recognizing that diet is a key environmental factor for modulation of gastrointestinal microbiota composition and metabolic function, and that the consumption of specific dietary ingredients such as fibers is an excellent form to benefit human gut microbiota and overall health since it is correlated with metabolic, immunologic, and protective functions in the human organism [54,55,56]. In this context, seaweeds are excellent sources of potential prebiotic fibers such as fucoidans, alginates, carrageenans, ulvans, and exopolysaccharides that are not digested, yet on reaching the colon are selectively fermented by beneficial colonic microbiota [56].

Total dietary fiber of tested seaweeds ranged from 15.0 g/100 g _dry seaweed_ in the red algae *G. gracilis* to 37.0 g/100 g _dry seaweed_ in the brown seaweed *F. vesiculosus*, with values being statistically different in all comparisons between species, except for comparisons between *P. palmata* and *U. rigida* (*p* < 0.05). Analysis of intra-species results shows that the brown and green algae, *F. vesiculosus* and *U. rigida*, possess total fiber contents higher than the three tested red algae species (*P. palmata*, *P. dioica*, and *G. gracilis)*. Regarding the soluble fiber contents, values ranged between 7.1 and 20.4 g/100 g _dry seaweed,_ both values being found within the red seaweed species, namely, *P. palmata* and *P. dioica*, respectively. Values were statistically different among all species (*p* < 0.05), except for comparisons between *G. gracilis* (red algae) and *U. rigida* (green algae) and *P. palmata* and *U. rigida*. The complete results are shown in Table 3. 

Dietary fiber of macroalgae contains several polysaccharides of high relevance for food, biomedical research, and therapeutic applications such as agars, ulvans, and fucoidans [57,58]. Sulfated polysaccharides, in particular, exhibit many biological activities such as antitumor, immunomodulatory, anticoagulant, and anti-mutagenic, among other relevant biological activities [5,7].

A very characteristic and highly relevant polysaccharide composition was found for each of the tested species. The brown seaweed *F. vesiculosus* revealed a total sugar content of 22%, which was mainly composed by uronic acids (UA, 42 mol%), mannose (Man, 21 mol%), and fucose (16 mol%) (Table 4). This composition is characteristic of *Fucus* seaweed, containing alginate (composed by mannuronic and guluronic acids) and fucoidan, composed by fucose and mannose [59,60]. *Ulva rigida* revealed a total sugar content of 28% composed mainly of glucose (Glc, 37 mol%) and containing a similar amount of rhamnose and uronic acid (23 mol% and 28 mol%, respectively), characteristic of ulvan, the main soluble polysaccharide of this seaweed [58]. These sulfated polysaccharides, as fucoidans and ulvans, have been widely researched due to their biological properties, namely, the immunomodulatory, hemostasis, pathogen inhibition, anti-inflammatory capacity, and antitumoral, being interesting compounds for application in health-related areas [61].

The red seaweed *P. palmata* had a lower total sugar content (9%), composed mainly by xylose (57 mol%) and galactose (22 mol%), with minor contents of UA, Glc, and Man (Table 4). This seaweed is reported to be constituted by mixed-linked xylans, xylogalactans, and acid xylomannans [60,61,62]. The total sugar content of *P. dioica* and *G. gracilis* were 30% and 27%, respectively. These seaweeds showed a sugar composition common for agar-producing red algae, with galactose as the main sugar residue (54 mol% and 34 mol%, respectively), as well as the presence of 3,6-anhydrogalactose (14 mol% and 25 mol%, respectively) and a naturally methyl-esterified sugar, 6-O-Me-galactose (11 mol% and 7 mol%, respectively) [63]. The complete results are shown in Table 4. Studies have been demonstrating the tremendous probiotic effects of galactose and some of its degradation products such as 3,6-anhydrogalactose and 6-O-Me-galactose [64,65]. In addition, 3,6-anhydrogalactose exhibited anti-cancer activity against human colon HCT-116 cells [65]. Galactose and 3,6-anhydrogalactose also demonstrated tremendous potential to be used as an ingredient for the cosmeceutical and food industries [66]. As demonstrated by Xie et al. (2020), polysaccharides from *Gracilaria chouae*, *Porphyra haitanensis*, and *Gracilaria blodgettii* as well as their degradation products showed dose-dependent tyrosinase inhibitory activity that ranged between 24.2% and 26.8% [66]. Furthermore, the degradation products showed a much higher tyrosinase inhibition than native polysaccharides [66].

### 2.4. Fatty Acid Composition 

Fatty acids comprise several important and diverse functions in cells functioning, which range from structural integrity of cell membranes to signaling molecules and suppliers of energy [67]. By influencing cell properties, fatty acids lead to the production of biologically active substances, altered metabolism, gene expression, and hormone responsiveness, influencing human health and well-being, physiological function, and disease risk [68]. In particular, Omega-3 and Omega-6 polyunsaturated fatty acids (PUFA) have been of special interest given their scientific evidenced benefits for overall human health and prevention of several chronic diseases [69,70,71,72]. The tested seaweeds revealed a qualitative profile that ranged between the saturated myristic fatty acid (C14:0) and omega-3 eicosapentaenoic acid (EPA; C20:5 n-3) with total fatty acid contents ranging from 4.10 µg/mg _dry seaweed_ in the red seaweed *P. palmata* to 26.39 µg/mg _dry seaweed_ in the brown seaweed *F. vesiculosus.* However, red and green macroalgae registered similar total fatty acid contents (4.10 to 6.99 µg/mg _dry seaweed_ in the red species versus 5.91 µg/mg _dry seaweed_ for the green *U. rigida*), and the brown macroalgae *F. vesiculosus* registered an almost four-fold higher content with 26.39 µg/mg _dry seaweed_ (Table 5). Palmitic acid (C16:0) was the predominant fatty acid among the three red macroalgae (*G. gracilis*, *P. palmata*, and *P. dioica*) and the green macroalga *U. rigida* (3.66, 1.99, 3.38, and 2.94 µg/mg _dry seaweed_, respectively), whereas for *F. vesiculosus*, although it also presented a considerable amount of palmitic acid (24.2% of total fatty acid content), its main compound was the monounsaturated oleic acid (C18:1 c9)—33.5% of total fatty acid content. These results agree with those found in scientific literature. Several studies have previously detected palmitic acid as the main component of several seaweed species [13,32,73,74,75,76]. Furthermore, Lorenzo et al. (2017) have previously characterized *F. vesiculosus* fatty acid profiles, pinpointing oleic and palmitic acids as its main components (19.94% and 14.66% of total fatty acid content, respectively) [27]. Furthermore, *F. vesiculosus* is distinct in its polyunsaturated fatty acid content, being the only source of eicosapentaenoic acid (EPA, C20:5 n-3) among the species studied, except for *P. palmata*, where five-fold lower content was detected.

The MUFA/SFA ratio ranged from 0.13 for the red macroalgae *P. dioica* to 0.85 for the brown macroalgae *F. vesiculosus*. Among the macroalgae species, the three red macroalgae *P. dioica*, *G. gracilis*, and *P. palmata*, presented the lowest MUFA/SFA ratios, followed by the green *U. rigida* and brown *F. vesiculosus* (Table 5). As expected, an inverse trend was observed for the SFA/MUFA ratio (Table 5). For the SFA/(MUFA + PUFAS) ratio, *F. vesiculosus* presented the lowest value with 0.81 followed by the green macroalgae *U. rigida* (1.81) and the three red macroalgae *G. gracilis*, *P. palmata*, and *P. dioica* (2.08, 2.18, and 5.50, respectively) (Table 5). In what concerns fatty acid composition, *F. vesiculosus* presents a more valuable nutritional profile in comparison with the other macroalgae studied. This brown macroalgae presents the highest MUFA/SFA ratio as well as the lowest SFA/MUFA and SFA/(MUFA + PUFAS) ratios. 

### 2.5. Total Phenolic Content, Free Radical Scavenging Activity, and Total Antioxidant Activity

Total phenolic content (TPC) of macroalgae ranged from 3.27 mg GAE/g _dry seaweed_ for the green seaweed *U. rigida* to 10.89 mg GAE/g _dry seaweed_ for the brown seaweed *F. vesiculosus*. Red seaweeds *P. palmata*, *G. gracilis*, and *P. dioica* presented higher total phenolic contents than the green counterparts but clearly below those presented by the brown species with 5.52, 4.41, and 3.87 mg GAE/g _dry seaweed_, respectively. Statistical differences in TPC (*p* < 0.05) were reported between *F. vesiculosus* and all the remaining species and between *U. rigida* and *P. palmata* (Table 6). The presence of phenolic compounds in macroalgae, which may include flavonols, catechins, flavonol glycosides, and in particular, phlorotannins (that can only be found in brown macroalgae species), have shown a noteworthy contribution to cancer, diabetes mellitus, and neurodegenerative and cardiovascular disease prevention, and continue to be the object of novel green extraction technologies and subsequent mechanistic and biological validation [5,77,78,79,80,81]. Currently, at least 301 metabolites harbored in marine macroalgae, including polyphenols, as well as sterols, carotenoids, vitamins, and several others, are known as a source of antioxidant activity [14,82,83]. It should be noted that this myriad of compounds are often multifunctional, expressing not only antioxidant or other previously mentioned biological properties but also technological traits (colorants, preservatives) to replace synthetic counterparts. This function versatility as well as high biodiversity increase the relevance of marine algae as good sources of such natural compounds for food and biomedical applications. This high interest is further supported by the advantageous growth conditions of algae and ease of handling, in comparison to other important plant-based sources of compounds with reported antioxidant activity, since not only are they are highly productive but they also do not need arable land for growth, contributing to sustainable production ecosystems [12,82]. 

**Figure 1:**
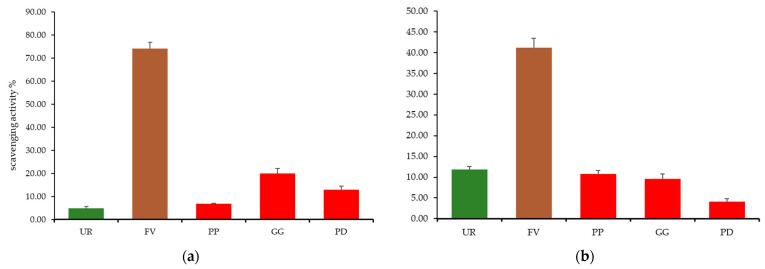
(**a**) DPPH free radical scavenging activity and (**b**) ABTS free radical scavenging activity of Ulva Rigida (UR), Fucus vesiculosus (FV), Palmaria palmata (PP), Gracilaria gracilis (GG), and Porphyra dioica (PD) seaweeds.

The total antioxidant and DPPH free radical activities of seaweed methanolic extracts are shown in Table 6. Interestingly, a good correlation between TPC of the tested seaweeds and corresponding antioxidant capacity is observed. The higher the TPC, the lower the DPPH antioxidant activity expressed as Trolox equivalents and the higher the percentage scavenging activity. *Ulva rigida*, which registered the lowest TPC, registered a DPPH activity of 0.129 mg Trolox equiv/g _dry seaweed_ (Table 6) and a percentage scavenging activity of only 9.7% (Figure 1), whereas *F. vesiculosus*, revealing the highest TPC showed, by far (at least four-fold), the lowest DPPH activity (0.033 mg Trolox equiv/g _dry seaweed_) and the highest percentage scavenging activity (74.1%). The tested red seaweed species showed intermediate values for both DPPH activity (Table 6) and percentage scavenging activity (Figure 1) which were statistically different (*p* < 0.05) from the brown species tested but similar between *P. palmata* and *P. dioica*, with *p* > 0.05. A similar tendency regarding the brown macroalgae highest antioxidant activity was observed by Tenorio-Rodriguez et al. (2017) when comparing 17 macroalgae ethanolic extracts (six *Rhodophyta*, four *Chlorophyta*, and seven *Ochrophyta*) collected at Baja California Peninsula, México [84]. Highest mean total phenolic content was registered for the brown macroalgae group (176.5 µg/GAE g dry weight), followed by red macroalgae (45.6 µg/GAE g dry weight) and green macroalgae (32.7 µg/GAE g dry weight) [84]. A similar tendency was observed regarding DPPH scavenging activity (higher values obtained for the brown macroalgae *Cystoseira osmundacea*—67.9%) and nitric oxide scavenging activity (higher values obtained for the brown macroalgae *E. arborea*, *P. concrecens*, *and D. delicatula*—reductions of 63.2%, 60.6%, and 59.8%, respectively), as well as for ferric reducing antioxidant power (FRAP) assay (brown macroalgae group had the highest value (4.6 ± 3.5 µM FeSO_4_ µg^−1^) followed by red macroalgae (3.1 µM FeSO_4_ µg^−1^) and green macroalgae (2.6 µM FeSO_4_ µg^−1^) [84].

In what concerns total antioxidant capacity measured by the ABTS assay, the values reported followed a similar trend to that observed for the DPPH scavenging capacity. *Fucus vesiculosus* registered a total antioxidant capacity of 8.20 mg Ascorbic Acid equiv/g _dry seaweed_ (Table 6), corresponding to a 40.3% ABTS scavenging activity. The remaining tested species revealed at least four-fold lower scavenging capacities. However, in this case, the green macroalgae *U. rigida* registered the second-highest scavenging activity value with 11.5%, followed by the three red macroalgae *P. palmata*, *G. gracilis*, and *P. dioica* with 10.6%, 9.3%, and 3.8%, respectively. *F. vesiculosus* results were similar to those reported for the brown macroalgae *Laminaria bongardiana* water extract (0.03 mg Ascorbic Acid/g_dry algae_) but lower than those obtained for both ethanolic or water extracts of other brown macroalgae species such as *Laminaria cichorioides*, *Kjellmaniella crassifolia*, *Undaria pinnatifida*, *Costaria costata*, and *Sargassum pallidum* [77]. In what concerns the red macroalgae species *P. dioica*, *G. gracilis*, *and P. palmata*, total phenolic content values were above those obtained for ultrasound-assisted extracts of *Gelidium sesquipedale* (ranging from 0.42 to 2.52 mg GAE/g dw) but lower regarding DPPH results (ranging from 0.35 to 0.49 Trolox equiv/g dw) [85]. Total phenolic content for the green *U. rigida* species were above those obtained for crude hydroalcoholic extract and crude aqueous fraction of the green macroalgae *Codium fragile* (2.202 and 0.298 mg GAE/g dw, respectively) but below those obtained for *Codium fragile* ethyl acetate fraction (22.381 mg GAE/g dw) and crude hydroalcoholic extract, ethyl acetate, and aqueous fractions of the green *Cladophora rupestris* (20.179, 21.726, and 15.833 mg GAE/g dw, respectively) [86].

With growing consumer demand and potential industrial applications, natural sources of antioxidant compounds such as the tested macroalgae herein are extremely valuable [16,19,87]. Furthermore, biomedical research has been associating macroalgae antioxidant capacity with benefits for several types of cancer, neurodegenerative diseases, and other pathologies [8,12,88,89,90,91]. 

## 3. Materials and Methods

### 3.1. Specimens of Seaweeds

Specimens of red algae, *Rhodophyta* (*Palmaria palmata*, *Porphyra dioica*, and *Gracilaria gracilis)*, green algae, *Chlorophyta* (*Ulva rígida*), and brown algae, *Phaeophyceae* (*Fucus vesiculosus*), were obtained from land-based fully controlled cultivation systems under the integrated multi-trophic aquaculture (IMTA) sustainable concept and provided by ALGAplus^®^—a company specialized in the production of seaweeds and their commercialization for food and cosmetic applications, based in Portugal (Aveiro district). The landbased open IMTA farming system of ALGAplus^®^ is located at the coastal lagoon of Ria de Aveiro. All macroalgae were harvested in January 2018. Algae taxonomic classification were based in the Algaebase [92]. All macroalgae were provided in dried powder with less than 1.0 mm particle size and were used for further experiments. 

### 3.2. Chemical Characterization of Seaweeds

#### 3.2.1. Proximate Composition

Moisture and ash contents were determined according to the AOAC methods (1990).

Total fat content was determined by Soxhlet extraction and nitrogen content by the Kjeldahl method, where protein is calculated by multiplying the nitrogen content by 6.25 [93,94]. Total sugar content was calculated by the sum of all monosaccharides determined, as described in Section 2.3. Total dietary fiber, insoluble dietary fiber, and soluble dietary fiber in seaweeds were determined by the method of Goering and Van Soest, 1975 [95]. 

#### 3.2.2. Elemental Composition 

Elemental composition was determined following the method described by Rodrigues et al. (2015) with some modifications [13]. First, a microwave-assisted acid digestion procedure was performed as proposed by Speedwave MW-3+ (Berghof, Berchtesgaden, Germany) for dried plant samples in dried seaweed samples. A sample with up to 0.25 g _dry seaweed_ was placed in the digestion vessel and added with 6 mL of concentrated nitric acid and 1 mL of hydrogen peroxide. The vessels were capped and placed in a microwave pressure digestor Speedwave MWS-3+ (Berghof) and subjected to microwave radiation at 20 bar according to the following program: room temperature was raised first to 130 °C at 22 °C/min and 30% of irradiation power, then to 160 °C at 6 °C/min and 40% of irradiation power, remaining 5 min at this temperature, and to 170 °C at 5 °C/min and 50% of irradiation power, remaining 5 min at this temperature. The cooling process consisted of decreasing temperature first to 100 °C for 4 min and then to room temperature. After cooling, acid digests were made up to 50 mL with Milli-Q water. Two replicates were performed for each seaweed sample as well as blanks. 

The content of each element is expressed as the mean plus standard deviation. The elemental composition was determined using an inductively coupled plasma (ICP) optical emission spectrometer model Optima™ 7000 DV ICP-OES (Dual View, PerkinElmer Life and Analytical Sciences, Shelton, CT, USA) with radial plasma configuration following the method previously described by Rodrigues et al. (2015). The accuracy of the method (microwave acid digestion and ICP-OES analysis) was assessed by analysis of certified reference material NIES-03 (Seaweed Chlorella; LGC standards, UK). Three replicates of reference material were subject to microwave digestion and analyzed three times by ICP-OES. Recovery ranged between 89% and 116%. 

To calculate individual molar Na:K ratios, contents of sodium and potassium (mg/10 g _dry seaweed_) of each sample were converted in millimoles (mmol) using the conversion 23 mg sodium = 1 mmol sodium and 39 mg potassium = 1 mmol potassium [42].

#### 3.2.3. Analysis of Fatty Acids

Fatty acid analysis was performed according to the procedure described by Fontes, Pimentel, Rodríguez-Alcalá, and Gomes, (2018), with some modifications [96]. To 100 mg seaweed sample, 200 µL of tritridecanoin (Sigma-Aldrich, St. Louis, MO, USA) was added. Then, 2.26 mL of methanol (Fisher Chemical, Fair Lawn, NJ, USA) and 800 µL of hexane (Sigma-Aldrich, St. Louis, MO, USA) followed by 240 µL of sodium methoxide (Sigma-Aldrich, St. Louis, MO, USA) were added. Samples were vortexed and incubated at 80 °C for 10 min. After cooling in ice, 1.25 mL of DMF (Sigma-Aldrich, St. Louis, MO, USA) and 1.25 mL of sulfuric acid (Fluka chemicals and reagents, Buchs, Switzerland) were added. Samples were vortexed and incubated at 60 °C for 30 min and 1 mL of hexane was added. Finally, after cooling, samples were vortexed and centrifuged (1250× *g*; 18 °C; 5 min). The upper layer containing methyl esters (FAME) was collected for further analysis. Samples were analyzed in a gas chromatograph HP6890A (Hewlett-Packard, Avondale, PA, USA) equipped with a flame-ionization detector (GLC-FID) and a BPX70 capillary column (60 m × 0.32 mm × 0.25 μm; SGE Europe Ltd., Courtaboeuf, France) following the chromatographic conditions described by Fontes et al. (2018) [97].

### 3.3. Polysaccharide Characterization 

Neutral sugar analysis was performed following the procedure described by Coimbra, Waldron, and Selvendran, [98], except for red macroalgae *Palmaria palmata*, *Porphyra dioica*, and *Gracilaria gracilis*, for which monosaccharide residues were obtained after acid reductive hydrolysis [99]. For all samples, the monosaccharides obtained after hydrolysis were derivatized to alditol acetates and analyzed by GC-FID, using 2-deoxyglucose (Sigma-Aldrich, St. Louis, MO, USA) as internal standard. Uronic acids (UA) were determined by a modification of the 3-phenylphenol colorimetric method [100].

### 3.4. Determination of Total Phenolic Content and Antioxidant Activity 

#### 3.4.1. Extract Preparation

Antioxidant extraction was performed according to Marinho, Sørensen, Safafar, Pedersen, and Holdt, 2019, with some modifications [101]. Each seaweed dried powder (1 g) was weighed into a centrifuge tube and 25 mL of methanol was added. Tubes were placed in a sonicator for 30 min. Each sample was centrifuged (2164× *g* for 10 min) and the supernatant was collected into a new tube. The pellet was resuspended, and the extraction process was repeated once. Extractions were performed for each of the three sampling replicates (n = 3). 

#### 3.4.2. Phenolic Content 

Determination of total phenolic content was performed by the method of Folin–Ciocalteu as previously described by Rodrigues, Sousa, et al., 2015, using gallic acid (LabChem, Zelienople, PA, USA) as standard [102]. Results are expressed as micrograms of gallic acid equivalents per gram of dry seaweed weight. The total polyphenol content of 2 mL was determined by colorimetry at 720 nm. Three true replicates were performed and analyzed in triplicate (n = 9). 

#### 3.4.3. DPPH Free Radical Scavenging Activity 

The DPPH free radical scavenging activity was determined according to the method described by Suresh et al., 2013, using Trollox (Sigma-Aldrich, St. Louis, MO, USA) as standard [103]. An aliquot (0.1 mL) of each extract (2 mg of lyophilized solids/mL) was added to 3.0 mL of a 0.1 mM ethanolic DPPH solution. After incubation for 30 min at 30 °C in the dark, the absorbance was measured at 517 nm. Three true replicates were performed and analyzed in triplicate (n = 9). Results were expressed as equivalent concentration of trollox (mg trollox equiv/g dry seaweed), whereas the percentage of scavenging activity was also calculated using the following formula:

$$Scavenging\%=\left(1-\frac{A_{sample}-A_{blank}}{A_{Control}}\right)\times 100$$

#### 3.4.4. Total Antioxidant Capacity

The total antioxidant capacity of extracts solutions was measured according to method described by Gião et al., 2007 [104]. To 2 mL of diluted ABTS solution 120 μL of extract was added and the absorbance at 734 nm of three true replicates was measured using ascorbic acid (Sigma-Aldrich, St. Louis, MO, USA) as standard; analyses were performed in triplicate. Results were expressed as equivalent concentration of ascorbic acid (mg ascorbic acid equiv/g dry seaweed). The percentage of scavenging activity was also determined using the following formula:

$$Scavenging\%=\left(\frac{A_{ABTS+}-A_{sample}}{A_{ABTS+}}\right)\times 100$$

### 3.5. Statistical Analysis 

Results are reported as mean values ± standard deviations. Statistical analysis was performed using IBM SPSS Statistics version 25 for Microsoft windows. Normality and homogeneity were examined and one-way ANOVA with the Holm-–Sidak test for post hoc analyses was applied to evaluate statistical differences between seaweeds (*p* < 0.05).

## 4. Conclusions

Results of the present study show that the tested macroalgae species, obtained from land-based fully controlled cultivation systems, possess an important nutritional profile, being a rich source of high-quality protein and dietary fiber, and providing considerable amounts of essential elements such as Ca, Mg, K, Mn, and Fe important in health promotion and disease prevention. Furthermore, these marine macroalgae are also an excellent source of different kinds of bioactive substances, including sulphated polysaccharides and phenolic compounds with significant antioxidant activity. Comparatively, among the five algae species tested, the brown macroalgae *F. vesiculosus* clearly stood out, presenting high polysaccharide content, rich in fucoidans and alginates, the highest phenolic content, as well as significant DPPH and ABTS scavenging activities. This study strongly contributes to generating an open mapping of land-based, integrated multi-trophic aquaculture (IMTA)-farmed seaweed composition. Furthermore, we demonstrated that the extraordinary nutritional and bioactive potential of these marine macroalgae can make them excellent ingredients for a wide range of food, cosmetic, and pharmaceutical industry applications. To further prove such potential, future studies should be conducted to explore the bioavailability and determine the in vivo bioactivity of these compounds, opening new research and industrial opportunities.

## Figures and Tables

**Table 1:** Proximate composition of seaweeds.

Parameter	*F. vesiculosus* 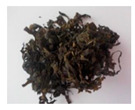	*G. gracilis* 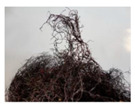	*P. palmata* 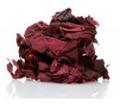	*P. dioica* 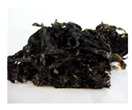	*U. rigida* 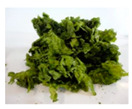
% moisture (g/100 g *)	12.4 ± 0.2 ^a^	4.1 ± 0.2 ^b^	3.2 ± 0.1 ^c^	10.4 ± 0.2 ^d^	16.2 ± 0.1 ^e^
% protein (g/100 g *)	12.4 ± 0.01 ^a^	28.8 ± 0.01 ^b^	41.8 ± 0.1 ^c^	35.7 ± 0.1 ^d^	19.5 ± 0.1 ^e^
% sugars (g/100 g *)	30.2 ± 1.3	26.8 ± 2.7	9.3 ± 0.1	29.8 ± 0.6	27.9 ± 0.4
% total fat (g/100 g *)	3.4 ± 0.2 ^a^	0.68 ± 0.01 ^b^	0.32 ± 0.02 ^b,c^	1.59 ± 0.01 ^d^	0.08 ± 0.004 ^c^
% ash (g/100 g *)	29.8 ± 0.1 ^a^	29.0 ± 0.2 ^a^	27.1 ± 0.6 ^b^	23.4 ± 0.03 ^c^	26.6 ± 0.4 ^b^

* Dry seaweed. a–e, in a row: different letters indicate significant differences (*p* < 0.05) between species.

**Table 2:** Elemental composition of seaweeds and their percentage impact on daily nutrient intake.

Elements	mg/ day	*F. vesiculosus* mg/10 g Portion	% R D I	*G. gracilis * mg/10 g Portion	% R D I	*P. palmata* mg/10 g Portion	% R D I	*P. dioica * mg/10 g Portion	% R D I	*U. rigida* mg/10 g Portion	% R D I
Calcium (Ca)	800	77.9 ± 1.6 ^a^	9.7	62.4 ± 0.3 ^b^	7.8	159.1 ± 1.4 ^c^	19.9	22.5 ± 2.3 ^d^	2.8	35.4 ± 2.5 ^e^	4.4
Potassium (K)	2000	369.6 ± 5.5 ^a^	18.5	892.0 ± 7.5 ^b^	44.6	345.2 ± 7.2 ^a^	17.3	286.3 ± 4.3^c^	14.3	136.4 ± 7.4 ^d^	6.8
Sodium (Na)	--	476.4 ± 7.6 ^a^	--	261.3 ± 1.7 ^b^	--	175.9 ± 4.2 ^c^	--	425.6 ± 6.2 ^d^	--	105.7 ± 3.3 ^e^	--
Magnesium (Mg)	375	81.3 ± 2.4 ^a^	21.7	40.2 ± 1.4 ^b^	10.7	22.20 ± 0.04 ^b^	5.9	61.7 ± 2.3 ^a,b^	16.5	186.0± ^c^	49.6
Phosphorus (P)	700	11.7 ± 0.2 ^a^	1.7	41.5 ± 1.7 ^b^	5.9	11.0 ± 0.18 ^a^	1.6	39.3 ± 1.4 ^c^	5.6	12.7 ± 0.2 ^d^	1.8
Iron (Fe)	14	10.3 ± 0.3 ^a^	73.6	16.5 ± 0.8 ^b^	117.9	4.6 ± 0.2 ^c^	32.9	6.2 ± 0.2 ^c^	44.4	13.1 ± 0.4 ^b^	93.6
Zinc (Zn)	10	0.56 ± 0.5 ^a^	5.6	0.41 ± 0.03 ^b^	4.1	0.18 ± 0.01 ^c^	1.8	0.73 ± 0.02 ^d^	7.3	0.14 ± 0.02 ^c^	1.4
Copper (Cu)	1	0.080 ± 0.001^a^	8.0	0.26 ± 0.01 ^b^	26.0	0.050 ± 0.001^a^	5.0	0.70 ± 0.03 ^c^	70.0	0.08 ± 0.01 ^a^	8.0
Manganese (Mn)	2	2.94 ± 0.03 ^a^	145.0	2.40 ± 0.03 ^b^	120.0	2.10 ± 0.02 ^c^	105.0	1.90 ± 0.01 ^c^	95.6	0.60 ± 0.02 ^d^	30.0
Na/K ratio (mmol)	--	2.19	--	0.50	--	0.86	--	2.52	--	1.31	--
Ca/Mg ratio (mg)	--	0.96	--	1.56	--	7.16	--	0.36	--	0.19	--

%RDI Based on daily intake portion of 10 g of dry seaweed. a–e, in a row: different letters indicate significant differences (*p* < 0.05) between species.

**Table 3:** Total, soluble, and insoluble dietary fiber contents of seaweeds.

Parameter		*F. vesiculosus*	*G. gracilis*	*P. palmata*	*P. dioica*	*U. rigida*
% fiber (g/100 g *)	Total	37.0 ± 0.4 ^a^	15.0 ± 0.03 ^b^	26.5 ± 0.7 ^c^	21.7 ± 0.8 ^d^	27.3 ± 1.6 ^c^
	Soluble	15.6 ± 1.1 ^a^	7.1 ± 0.1 ^b^	9.8 ± 0.4 ^c^	20.4 ± 0.9 ^d^	8.9 ± 1.4 ^b,c^
	Insoluble	21.4 ± 1.6 ^a^	7.9 ± 0.2 ^b^	16.7 ± 0.8 ^c^	1.3 ± 0.05 ^d^	18.4 ± 0.2 ^c^

* Dry seaweed. a–d in a row: different letters indicate significant differences (*p* < 0.05) between species.

**Table 4:** Monosaccharide composition (mol%) for all seaweed samples analyzed.

mol %	*F. vesiculosus*	*U. rigida*	*P. palmata*	*P. dioica*	*G. gracilis*
Rha	0.4	23.1	0.3	0.1	0.5
Fuc	14.3	--	2.3	0.1	0.7
Rib	2.1	0.2	1.1	0.7	0.6
Ara	0.4	--	0.2	0.2	--
3,6-AnGal	--	--	0.2	14.4	24.6
Xyl	2.0	6.5	56.5	6.5	2.0
6-O-Me-Gal	--	--	--	10.9	7.3
4-O-Me-Gal	--	--	--	--	4.5
Man	17.2	1.3	3.3	7.4	0.4
Gal	3.0	3.3	21.9	53.8	34.1
Glc	9.4	37.3	6.0	2.6	19.3
UA	51.2	28.4	8.1	3.3	5.9

Ara—arabinose, Fuc—fucose, Gal—galactose, Glc—glucose, Man—manose, Rha—rhamnose, Rib—ribose, UA—uronic acid, Xyl—xylose, 3,6-AnGal—3,6-anhydrogalactose, 4-O-Me-Gal—4-O-Me-galactose, 6-O-Me-Gal—6-O-Me-galactose.

**Table 5:** Fatty acid composition (µg/mg of dry weight) and total saturated, monounsaturated, and polyunsaturated fatty acid fractions of each seaweed species.

	Seaweed	*F. vesiculosus*		*G. gracilis*		*P. palmata*		*P. dioica*		*U. rigida*	
Fatty Acids		Mean	SD	Mean	SD	Mean	SD	Mean	SD	Mean	SD
**C14i**		0.29 ^a^	0.01	0.18 ^b,c^	0.02	0.12 ^c^	0.01	0.23 ^a,b^	<0.01	0.24 ^a,b^	0.02
**C14**		3.76 ^a^	0.03	0.71 ^b^	0.01	0.54 ^c^	0.01	0.80 ^b^	0.01	0.08 ^d^	0.00
**C15**		0.17	<0.01	--	--	--	--	--	--	--	--
**C15:1**		0.20 ^a^	0.01	0.13 ^a,b^	0.01	0.09 ^b^	0.01	0.17 ^a,b^	<0.01	0.18 ^a,b^	0.02
**C16**		6.39 ^a^	0.06	3.66 ^b^	0.02	1.99 ^c^	0.06	3.38 ^b,c^	0.03	2.94 ^c^	0.21
**C16:1**		--	--	--	--	--	--	0.16	<0.01	--	--
**C16:1 c7**		0.12 ^a^	<0.01	0.08 ^b^	0.01	0.16 ^c^	<0.01	--	--	--	--
**C16:1 c9**		0.45 ^a^	<0.01	0.58 ^b^	0.01	--	--	--	--	--	--
**C17**		0.09 ^a^	<0.01	--	--	--	--	0.65 ^b^	0.01	0.27 ^c^	0.02
**C17:1 c10**		0.09 ^a^	<0.01	--	--	--	--	--	--	0.24 ^b^	0.02
**C18**		0.71 ^a^	0.01	0.16 ^b^	0.01	0.15 ^b^	0.01	0.11 ^c^	<0.01	0.14 ^b^	0.04
**C18:1 t4**		0.21 ^a^	<0.01	--	--	--	--	--	--	0.16 ^a^	0.01
**C18:1 c9**		8.83 ^a^	0.05	0.66 ^b^	<0.01	0.71 ^b^	0.04	0.14 ^c^	<0.01	--	--
**C18:1 c11**		0.17 ^a^	<0.01	0.22 ^b^	<0.01	0.17 ^a^	0.01	0.21 ^b^	<0.01	1.22 ^c^	0.11
**C18:2 c9c12**		1.38 ^a^	<0.01	0.10 ^b^	<0.01	0.07 ^b^	0.01	--	--	0.12 ^b^	0.02
**C18:3 c9c12c15**		0.91 ^a^	0.01	--	--	--	--	--	--	0.18 ^b^	0.03
**C20**		0.17 ^a^	<0.01	--	--	--	--	--	--	0.13 ^b^	0.02
**C18:2 c9t11**		0.07 ^a^	<0.01	--	--	--	--	0.18 ^b^	<0.01	--	--
**C21**		0.08	<0.01	--	--	--	--	--	--	--	--
**C20:4 n6**		1.63 ^a^	0.01	0.50 ^b^	0.05	--	--	0.07 ^c^	<0.01	--	--
**C22**		0.14 ^a^	<0.01	--	--	--	--	--	--	--	--
**C20:5 n3**		0.53 ^a^	<0.01	--	--	0.10 ^b^	0.01	--	--	--	--
**Total µg/mg (dry seaweed)**		26.39		6.99		4.10		6.11		5.91	
**SFA**		11.79		4.72		2.81		5.17		3.80	
**MUFA**		10.08		1.66		1.12		0.68		1.80	
**PUFAS**		4.52		0.61		0.17		0.26		0.30	
**MUFA/SFA ratio**		0.85		0.35		0.40		0.13		0.47	
**SFA/MUFA ratio**		1.17		2.84		2.51		7.60		2.11	
**SFA/(MUFA + PUFAS) ratio**		0.81		2.08		2.18		5.50		1.81	

Data expressed as mean (Mean; *n* = 2) and standard deviation (SD). SFA/MUFA/PUFA: total of saturated/monounsaturated/polyunsaturated fatty acids. a–c: in a row, different letters mean significant differences among seaweed species (*p* < 0.05).

**Table 6:** Total phenolic content, radical scavenging activity, and total antioxidant capacity of methanolic extracts from tested algae species.

	Radical Scavenging Activity	Total Antioxidant Capacity	Total Phenolic Content
**SEAWEED**	**DPPH**, mg Trolox equiv/g Dry Algae Extract	**ABTS**, mg Ascorbic Acid equiv/g Dry Algae Extract	**Folin–Ciocalteu**, mg Gallic acid equiv/g % Dry Algae Extract
***F. vesiculosus***	0.033 ± 0.002 ^a^	8.20 ± 0.07 ^a^	10.89 ± 0.61 ^a^
***U. rigida***	0.129 ± 0.002 ^b^	9.89 ± 0.70 ^b^	3.27 ± 0.25 ^b^
***P. palmata***	0.127 ± 0.010 ^b^	8.35 ± 0.20 ^a^	5.52 ± 0.52 ^c^
***P. dioica***	0.126 ± 0.003 ^b^	9.67 ± 0.06 ^b^	3.87 ± 0.41 ^b,c^
***G. gracilis***	0.115 ± 0.005 ^c^	8.90 ± 0.11 ^c^	4.41 ± 0.21 ^b,c^

a–c: in a row, different letters mean significant differences among seaweed species (*p* < 0.05).

## Data Availability

The data are available from the corresponding author.

## Sample Availability

Samples of the compounds are available from the authors.
